# Adverse effects in children exposed to maternal HIV and antiretroviral therapy during pregnancy in Brazil: a cohort study

**DOI:** 10.1186/s12978-018-0513-8

**Published:** 2018-05-10

**Authors:** Adriane M. Delicio, Giuliane J. Lajos, Eliana Amaral, Fernanda Cavichiolli, Marina Polydoro, Helaine Milanez

**Affiliations:** 1 Department of Obstetrics and Gynecology, School of Medical Sciences, University of Campinas, Campinas, Brazil; 2 Department of Clinics, School of Medical Sciences, University of Campinas, Campinas, Brazil; 3 Referral Center for STIs and AIDS of Campinas, Campinas, Brazil

**Keywords:** HIV, Toxicity, Adverse effects, Antiretroviral therapy, Pregnancy, Newborn, HIV, Toxicidade, Efeitos adversos, Terapia antirretroviral, Gestação, Recém-nascido

## Abstract

**Background:**

Antiretroviral therapy (ART) in pregnancy was associated with a drastic reduction in HIV mother-to-child transmission (MTCT), although it was associated with neonatal adverse effects. The aim of this study was to evaluate the neonatal effects to maternal ART.

**Methods:**

This study was a cohort of newborns from HIV pregnant women followed at the CAISM/UNICAMP Obstetric Clinic from 2000 to 2015. The following adverse effects were evaluated: anemia, thrombocytopenia, liver function tests abnormalities, preterm birth, low birth weight and congenital malformation. Data collected from patients’ files was added to a specific database. Descriptive analysis was shown in terms of absolute (n) and relative (%) frequencies and mean, median and standard deviation calculations. The association between variables was tested through Chi-square or Fisher exact test (*n* < 5) and relative risk (RR) with its respective *p* values for the categorical ones and *t*-Student (parametric data) or Mann-Whitney (non-parametric data) for the quantitative ones. The significant level used was 0.05. A multivariate Cox Logistic Regression was done. Statistical analysis was performed using SAS version 9.4.

**Results:**

Data from 787 newborns was analyzed. MTCT rate was 2.3%, with 0.8% in the last 5 years. Observed neonatal adverse effects were: liver function tests abnormalities (36%), anemia (25.7%), low birth weight (22.5%), preterm birth (21.7%), children small for gestational age (SGA) (18%), birth defects (10%) and thrombocytopenia (3.6%). In the multivariate analysis, peripartum CD4 higher than 200 cells/mm^3^ was protective for low birth weight and preterm birth, and C-section was associated with low birth weight, but not with preterm birth. Neonatal anemia was associated with preterm birth and exposure to maternal AZT. Liver function tests abnormalities were associated with detectable peripartum maternal viral load and exposure to nevirapine. No association was found between different ART regimens or timing of exposure with preterm birth, low birth weight or congenital malformation.

**Conclusion:**

Highly active antiretroviral treatment in pregnant women and viral load control were the main factors associated with MTCT reduction. Antiretroviral use is associated with a high frequency but mainly low severity adverse effects in newborns.

## RESUMO

**Introdução:**

O uso da terapia antirretroviral (TARV) na gestação se associou a dramática redução da transmissão vertical (TV) do HIV, porém demonstrou poder estar relacionado a efeitos adversos neonatais. O objetivo deste estudo foi avaliar os efeitos neonatais decorrentes da exposição à TARV materna.

**Métodos:**

estudo observacional analítico de uma coorte de recém-nascidos de gestantes infectadas pelo HIV atendidos no Serviço de Obstetrícia do CAISM/UNICAMP entre 2000 e 2015. Foram avaliados os seguintes efeitos adversos: anemia, plaquetopenia, alteração hepática, prematuridade, baixo peso e malformação congênita. Os dados foram coletados dos prontuários dos pacientes e inseridos em banco específico. A análise descritiva foi realizada por meio de frequências simples (n) e relativas (%) e cálculos de média, desvio-padrão e mediana. As associações entre variáveis foram testadas por meio do Qui-quadrado ou Exato de Fisher (*n* < 5) e Razão de Risco com respectivo valor de *p* para as categóricas e por meio do *t* Student (dados paramétricos) ou Mann-Whitney (não-paramétricos) para as quantitativas. O nível de significância foi de 0,05. A análise multivariada foi realizada através da Regressão Logística de COX. No processamento e análise dos dados, foi utilizado o programa SAS 9.4.

**Resultados:**

foram analisados dados de 787 recém-nascidos. A taxa de TV do HIV foi de 2,3%, sendo 0,8% nos últimos 5 anos. Os efeitos adversos observados foram alteração hepática (36%), anemia (25,7%), baixo peso (22,5%), prematuridade (21,7%), crianças pequenas para idade gestacional (PIG) (18%), malformações congênitas (10%) e plaquetopenia (3,6%). Em análise multivariada, o CD4 periparto maior que 200 células/mm^3^ foi protetor para baixo peso e prematuridade, e a cesárea esteve associada ao baixo peso ao nascimento, mas não ao parto prematuro. A anemia esteve associada ao parto prematuro e à exposição a zidovudina materna. A alteração hepática esteve associada à carga viral materna periparto detectável e à exposição a nevirapina. Não houve associação entre diferentes esquemas de TARV e tempo de exposição às drogas maternas com prematuridade, baixo peso e malformação congênita.

**Conclusão:**

a TARV potente materna com consequente controle da carga viral é o maior fator responsável pela redução da TV do HIV. Ela está associada a frequência elevada de efeitos adversos no recém-nascido, porém a maioria de menor gravidade.

## Plain English summary

HIV treatment during pregnancy controls maternal disease and reduces the risk of transmitting the virus to the baby. However, medication can cause some adverse effects in newborns such as anemia and liver changes, besides low birth weight or birth before the baby is completely mature. Our study was an investigation about the impact of the antiretroviral therapy over infants’ health. We studied information on 787 children born from HIV positive women between 2000 and 2015 at the Women’s Hospital at UNICAMP. After data analysis, we observed that the HIV transmission to the baby was low, especially in the last five years of the research. We also found a high occurrence of liver changes (36%), anemia (25.7%), low birth weight (22.5%), premature babies (21.7%) and birth defects (10%). Of these findings, prematurity, low birth weight or birth defects were not caused by the medication used during pregnancy. On the other hand, anemia and liver changes in the babies could have been caused by the mothers’ medication. From our study we concluded that, despite the high occurrence of side effects in the children born from mothers who tested positive for HIV, they are not severe, confirming the need for mother to take medication during pregnancy so the children can be born free from HIV.

## Background

Around 36.7 million people are infected with human immunodeficiency virus (HIV) in the world. Of the 1.8 million new infections in 2016, almost half were women and 160,000 were children under 15 years who were infected through mother-to-child transmission (MTCT) [1].

MTCT can occur during pregnancy, delivery or breastfeeding. With no intervention MTCT risk was near 40% [2, 3]. Antiretroviral therapy (ART) for pregnant women is the best intervention to reduce the risk of infection to rates lower than 2%, increasing maternal and infant life expectancy [4, 5].

Through the years, the monotherapy with zidovudine recommended in the 1994 ACTG 076 protocol changed to a 3-drug antiretroviral therapy (ART). World Health Organization (WHO) treatment guidelines in 2013 recommended the universal use of ART in HIV infected pregnant women (B+ Option), independently of the CD4 count or the diseases status [6, 7]. ART should be maintained after birth to control maternal disease and to prevent MTCT and sexual HIV transmission (7). The treatment to HIV pregnant women follows the guidelines to ART in HIV infected adults [8].

The effects on the child and the ART safety use in the mother has been demonstrated through the years. Despite the unquestionable benefits of ART in reducing MTCT of HIV and the differences in the placental and pharmaco-kinetic transference of ART during pregnancy, every drug carries a potential risk for adverse effects [9]. The progressive presence of maternal antiretroviral in meconium with the increased gestational age demonstrates the fetal exposure to these drugs [10]. The adverse effects described in most of the studies are mainly hematological, hepatic and mitochondrial changes, preterm birth and low birth weight, neonatal mortality, fetal growth restriction, congenital malformation and viral resistance [11–14].

The Women’s Hospital at University of Campinas School of Medical Sciences (CAISM/UNICAMP) has maintained a program for pregnant women infected with HIV since 1988, and has accumulated great experience in the use of ART. The aim of this study was to evaluate adverse hematologic and hepatic effects, preterm birth and low birth weight in a cohort of infants whose mothers had HIV infection and were followed at the Obstetrics Clinic in a public university hospital in Brazil.

## Methods

Observational analytic study based on the evaluation of a historic cohort of pregnant women infected with HIV and their exposed newborns seen at the Obstetrics Clinic at the University of Campinas School of Medical Sciences (CAISM/UNICAMP) between 2000 and 2015. CAISM is the reference hospital for high risk pregnancy in Campinas, the second biggest city in the State of São Paulo with 1.2 inhabitants and an Human Development Index (HDI) of 0.8. Mostly, CAISM serves pregnant women without health insurance and from low socioeconomic status. Data about the women and their newborns was collected from the clinical files and from the epidemiologic Health Surveillance Agency. A specific form was developed to collect all the information.

The following variables were analyzed: pregnant women’s epidemiological and clinical characteristics, regimens of ART used, mode of delivery, MTCT of HIV, newborn characteristics (weight, height, age by Capurro, Apgar, birth defects, neonatal disease), use of post-natal prophylaxis and newborn’s laboratorial results (anemia, thrombocytopenia, liver function tests abnormalities). Neonatal death was defined as death of a live birth up to 28 days and fetal death as birth with no signs of life after the 20^th^ week of gestational age. Time of ART introduction was described in gestational weeks; women in use of ART in the last menstrual period were considered in treatment at conception. The combination of antiretroviral drugs considered for the global analysis were: monotherapy with zidovudine (AZT); double therapy with nucleoside reverse transcriptase inhibitors (NRTI) and combined therapy (ART). Combined therapy was defined as a combination of at least three drugs with no less than one protease inhibitor (PI) or one non-nucleoside reverse transcriptase inhibitor (NNRTI). Women using the PI darunavir (DRV), lopinavir (LPV) and atazanavir (ATV) also received booster of ritonavir (R).

To evaluate the adverse effects of antiretroviral medication in the newborns the main outcomes were: preterm birth (defined as gestational age under 37 weeks or very preterm birth under 34 weeks), low birth weight (defined as birth weight less than 2500 g or very low birth weight under 1500 g), and children small for gestational age (SGA) [15]. Abnormal laboratory results included anemia (hemoglobin < 13,5 g/dl), thrombocytopenia (platelets < 150,000/ml), liver function tests abnormalities (at least one: alanine aminotransferase-ALT > 31 U/l, aspartate aminotransferase-AST > 122 U/l, alkaline phosphatase-ALCPH > 250 U/l, gamma-glutamyl transferase-GGT > 151 U/L, bilirubin > 1,0 mg/dl) [16].

All newborns exposed to HIV infection were followed at the Pediatric Immunodeficiency Service at the same University Hospital. The cases with no final HIV infection diagnosis due to loss of follow-up were contacted by phone. This study was approved by the Institution’s Ethics in Research Committee (protocol #351/2006).

Descriptive analysis was shown in terms of absolute (n) and relative (%) frequencies, mean, median and standard deviation. Chi-square or Fisher exact test (*n* < 5) was used to analyze the association between the categorical variables. For the continuous variables, the Student *t*-test (parametric data) or Mann-Whitney (non-parametric data) were used. The specific effects of the different antiretroviral regimens were analyzed using relative risk (RR) with its respective *p* values. A multivariate Cox Logistic Regression analysis was done. A 95% confidence interval (CI) and a significant level of 0.05 were used. Statistical analysis was performed using SAS version 9.4.

## Results

**Fig. 1:**
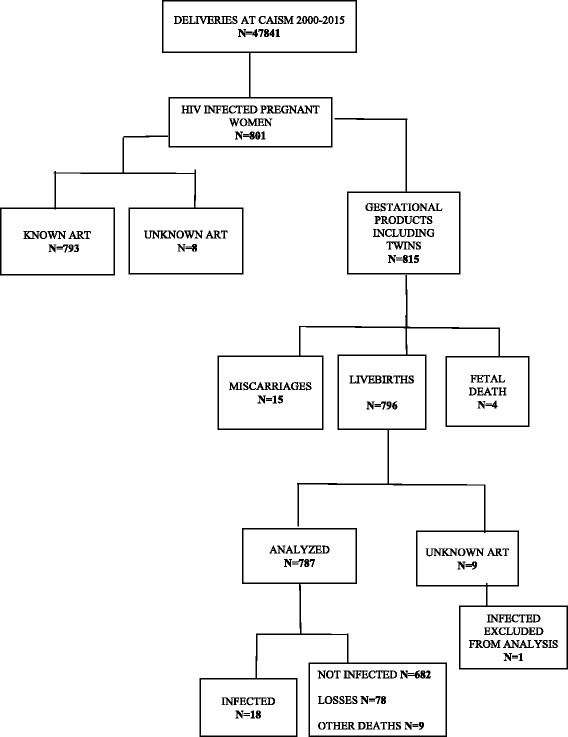
Fluxogram of pregnant women infected with HIV and exposed children between 2000 and 2015. ART antiretrovitral therapy

Between 2000 and 2015, 47,841 births occurred at the site where this study took place. From these, 801 were pregnant women infected with HIV, with a 1.67% prevalence rate. Figure 1 shows all the eligible cases, dropped patients and final newborn numbers included in the analysis (*n* = 787). The MTCT rate was 2.3%. In the last 5 years, of the 350 infected pregnant women, there were only three cases of HIV transmission, all of them from women with low adherence to ART and substance abuse.

### Pregnant women, prenatal care and delivery characteristics

The mean age of the pregnant women was 28 years; 61.2% were white, 60.9% completed elementary school and the median parity was one child. Pregnancy was planned in 25.7% of the cases. Exposure to HIV was heterosexual in 93% of the women and 10 of them (2.5%) had acquired the infection through MTCT.

Sixty-two percent of the women knew about their HIV status before the pregnancy, 47% had taken some antiretroviral drug previously and 27% were using ART at conception. The most used combination was the two NRTI and one PI or one NNRTI, with 81 (10.2%) using Efavirenz (EFV) at conception. The median gestational week at the start of prenatal care was 17.1 weeks and the mean number of prenatal visits was 7.6.

A little more than half (52.7%) of the women presented at least one of the following gestational complications: substance abuse (crack) in 14.3%, smoking (14.3%), preterm labor (14.2%), hypertension (7.4%), alcoholism (5.8%), intrauterine grow restriction (4.4%) and psychiatric problems (2.9%), among others. At least 767 women (84.8%) presented at least one infection: urinary infection (34.9%), bacterial vaginosis (33.8%), *Streptococcus* B colonization (33.4%), intracervical papillomavirus/neoplasia (14.3%), hepatitis C (7.6%), latent tuberculosis (5,6%), syphilis (5.2%), genital herpes (2.2%), active tuberculosis (1.7%), and hepatitis B (0.4%). Only four patients presented multidrug resistance (3.3%).

Fifty-one percent of the pregnant women were classified into the CDC stage 2 and 18.5% were classified as having Acquired Immunodeficiency Syndrome (AIDS). Only 32 women (4.1%) presented opportunistic infections during pregnancy. Fifteen (1.9%) women did not use antiretroviral therapy while pregnant because the HIV diagnosis was done during labor. The 81 patients in use of efavirenz in the first trimester had the drug changed to PI during the prenatal care, except for one which maintained it throughout pregnancy. Only four pregnant women were using EFV towards the end of gestation, including three which started EFV in the second trimester. Twenty-three (2.9%) women used monotherapy with AZT and 11 (1.4%) used the double therapy (zidovudine e lamivudine). Most of the pregnant women used combined ART: 17% with two NRTI and nevirapine (NVP), 17% with two NRTI and nelfinavir (NFV), 54% with two NRTI and lopinavir/ritonavir (LPV/R), 5% with two NRTI and other PI (26 with ATV/R, 6 with indinavir, 3 with darunavir, 3 with saquinavir, and 2 with fosamprenavir). Five women used a combination of two NRTI with NVP and PI simultaneously (3 with LPV/R, 1 with ATV/R and 1 with NFV, included in their respective groups). The most used NRTI were zidovudine (AZT) and lamivudine (3TC). AZT was changed to tenofovir (TDF) in 41 cases, to stavudine in seven cases and to abacavir in one case. The AZT combination with TDF cases (17 patients) were excluded from the specific analysis. The integrase inhibitor raltegravir (RAL) was added to the ART regimen in seven cases (four cases included in the LPV/R group and three cases with DRV/R, included in the Regimens with other PI group), mostly in the late gestational weeks (Table 1).

**Table 1:** Characteristics of pregnant women infected with HIV at CAISM/UNICAMP from 2000 to 2015

Characteristics	n	Median (%)
Age (years)	793	28 (13–46)
Parity	793	1 (0–9)
Schooling years (*n* = 769)		
< 8	468	60.9
8–11 yrs.	248	32.2
> 12	39	5.1
No schooling	14	1.8
Ethnicity: White	485	61.2
HIV diagnosis (*n* = 789)		
Prior to pregnancy	489	62.5
During pregnancy	294	37.5
In use of ART at conception (*n* = 787)	217	27.6
In use of Efavirenz at conception (*n* = 793)	81	10.2
Planned pregnancy (*n* = 382)	98	25.7
Heterosexual transmission (*n* = 408)	380	93.1
Opportunistic disease during pregnancy (*n* = 785)	32	4.1
First CD4 median (cel/ml)	734	444 (3–1915)
Peripartum CD4 median (cel/ml)	175	552 (5–2164)
Median time of ART use (days)	410	152.5 (4–292)
Peripartum Viral Load < 50 (copies/ml) (*n* = 732)	432	58.9
CDC classification (*n* = 758)		
1	227	29.9
2	391	51.6
3	140	18.5
Coinfections during pregnancy (*n* = 787)	667	84.8
Obstetrics complications (*n* = 792)	417	52.7
ART adherence during prenatal care	649	84.3
Changed ART (*n* = 776)	149	19.2
Premature rupture of membranes (*n* = 773)	121	15.7
Labor (*n* = 447)	188	42.1
Intravenous AZT (*n* = 775)	735	94.8
Antiretroviral regimens during pregnancy (*n* = 793)		
None	15	1.9
Monotherapy with AZT	23	2.9
NRTI + NRTI	11	1.4
NRTI + NRTI + NVP	138	17
NRTI + NRTI + NFV	135	17
NRTI + NRTI + LPV/R	428	54
Regimens with other PI	40	5
NRTI + NRTI + EFV	3	0.4
TOTAL	793	100

*ART* antiretroviral therapy, *AZT* zidovudine, *NRTI* nucleos(t)ide reverse transcriptase inhibitors, *NNRTI* non-nucleoside reverse transcriptase inhibitor, *NVP* nevirapine, *EFV* efavirenz, *PI* protease inhibitor, *NFV* nelfinavir, *LPV* lopinavir, *R* ritonavir, *CDC* Centers for Disease Control and Prevention

The median time of antiretroviral therapy during pregnancy was 152.5 days. The median initial CD4 was 444 cells/ml (varying from 3 to 1915) and the median perinatal count was 552 cells/ml. The median viral load in the first count was 1371 copies/ml, with 432 (58.9%) women with undetectable viral load in the end of the pregnancy.

Intravenous AZT was used in 94.8% of the cases. Cesarean section was done in 92.8% of cases, mostly due to the HIV infection, according to the hospital guidelines between 2005 to 2015. There were only four episiotomies in the vaginal births and 42.1% of women went into labor, with 15.7% having premature rupture of the membrane.

### Newborn characteristics

Of the 787 newborns, 384 (50.7%) were females. The birth weight and the mean length were 2850 g and 47 cm respectively. The median age at birth by the Capurro method was 38 weeks. One hundred and sixty-seven (21.7%) were preterm and 2.9% were very preterm birth. There were 22.5% low birth weight newborns, 3.6% with very low birth weight and 18% small for gestational age (SGA). Ninety-nine percent received an Apgar higher than 7 in the 5th minute.

All newborns received neonatal AZT oral prophylaxis. During follow-up, 81.3% received AZT for six weeks and 17.1% for four weeks. Only one newborn did not use prophylactic medication after hospital discharge. Twenty-four newborns (3.1%) received nevirapine associated with AZT in the first week of life to maximize the neonatal prophylaxis. Despite dispensation of cabergoline immediately after birth to every new mother and the recommendation to suspend breastfeeding, two newborns (0.3%) were breastfed.

Two hundred and nine (27.2%) newborns presented at least one disease in the neonatal period. The most common neonatal complications were: respiratory distress (15), neurologic disorders (14), newborn transitory tachypnea (11), hyaline membrane disease (5), neutropenia (5), hypoglycemia (5), withdrawal syndrome from maternal substance abuse (3), hypothyroidism (3) and myocarditis associated to ART (1). The most frequent infectious diseases were: syphilis (13), pneumonia (6), sepsis (5), cytomegalovirus (4), toxoplasmosis (3), meningitis (3), hepatitis C (2), varicella (1) and urinary tract infection (1).

Seventy-nine (10%) children presented congenital malformations: 23 in the nervous system, 21 had cardiopathy, 20 had hemangiomas and other skin problems, 12 in the urinary tract, 11 had muscle and skeletal anomalies, 3 had genital malformations, 3 with genetic anomalies and 2 had gastrointestinal congenital problems.

As for adverse effects, 36% of the children presented liver function tests abnormalities, 25.7% had anemia and 3.6% had thrombocytopenia at birth.

### Association between prenatal characteristics and neonatal adverse effects

Low birth weight was associated with hypertension (RR 2.01 CI 1.26–3.2), smoking (RR 1.73 CI 1.12–2.67), substance abuse (RR 1.61 CI 1.04–2.48) and C-section in multivariate analysis (RR 1.96 CI 1.09–3.52). CD4 higher than 200 cells/mm^3^ was protective for low birth weight (RR 0.47 CI 0.29–0.77) (Table 2).

Preterm birth was associated with maternal AIDS (RR 1.80. CI 1.20–2.7), but not associated with cesarean (RR 1.43 CI 0.85–2.39). CD4 higher than 200 cells/mm^3^ was protective for preterm birth (RR 0.54 CI 0.37–0.79) (Table 2).

In the multivariate analysis, neonatal anemia was associated with preterm birth (RR 1.44 CI 1.04–1.99). The occurrence of liver function tests abnormalities was associated with perinatal maternal detectable viral load (RR 2.37 CI 1.52–3.68) (Table 2).

**Table 2:** Comparative analysis between children exposed to NNRTI and PI at CAISM/UNICAMP from 2000 to 2015

Variables	NNRTI		PI		p*	RR	CI 95%
	*n*	%	*n*	%			
Gestational age (Capurro)					0.2212		
<37 weeks	25	17.7	130	22.5		0.79	0.53–1.16
>=37 weeks	116	82.3	449	77.5		1	
Birth weight					0.4214		
<2500 g	28	19.7	135	22.8		0.86	0.59–1.25
>=2500 g	114	80.3	456	77.2		1	
Weight adequacy for GA					0.0051		
Adequate for GA	126	89.4	449	77.1		1	
Small for GA	14	9.9	118	20.3		0.48	0.29–0.81
Large for GA	1	0.7	15	2.6			0.04–1.91
5th minute Apgar					0.9752		
<7	1	0.7	4	0.7		1.03	0.18–5.97
>=7	141	99.3	584	99.3		1	
Neonatal disease					0.4002		
Yes	33	24.1	160	27.6		0.86	0.60–1.23
No	104	75.9	419	72.4		1	
Birth defect					0.5148		
Yes	12	8.5	61	10.3		0.84	0.49–1.44
No	130	91.5	533	89.7		1	
Anemia					0.0403		
Yes	39	32.2	129	23.3		1.43	1.02–2.01
No	82	67.8	424	76.7		1	
Thrombocytopenia					0.2841		
Yes	2	1.7	22	4		0.46	0.12–1.74
No	119	98.3	532	96		1	
Hepatic changes					< 0.0001		
Yes	21	80.8	100	31.4		7.74	2.99–20.01
No	5	19.2	218	68.6		1	
TOTAL	142	100	594	100			

*GA* gestational age, *NNRTI* non-nucleoside reverse transcriptase inhibitor, *PI* protease inhibitor

(#) Numbers are different due to the lack of information on some patients

*Chi-square

### Association between maternal ART exposure and neonatal adverse effects

Use of NVP showed a higher occurrence of anemia (*p* = 0.0403, RR 1.43 CI 1.02–2.01) and liver function tests abnormalities (*p* < 0.0001, RR 7.74 CI 2.99–20.01) at birth when compared to the PI use (Table 3).

**Table 3:** Comparative analysis between children exposed to different protease inhibitors at CAISM/UNICAMP from 2000 to 2015

Variables	ATV		NFV		LPV/R		p*	ATV x LPV/R		NFV x LPV/R		ATV x NFV	
	*n*	%	*n*	%	*n*	%		RR	CI 95%	RR	CI 95%	RR	CI 95%
Gestational age (weeks)													
<34 weeks	0	0	4	3.1	11	2.7	0.0922	NC		0.71	0.47–1.07	NC	
>=34 weeks	25	100	126	96.9	402	97.3				1.00			
Gestational age (Capurro)													
<37 weeks	6	24	22	16.9	99	24	0.0922	1.00	0.41–2.44	0.71	0.47–1.07	1.43	0.63–3.26
>=37 weeks	19	76	108	83.1	314	76		1.00		1.00		1.00	
Birth weight													
<2500 g	5	19.2	24	18.2	104	24.6	0.1242	0.74	0.29–1.92	0.74	0.50–1.10	1.06	0.44–2.57
>=2500 g	21	80.8	108	81.8	318	75.4		1.00		1.00		1.00	
Birth weight													
<1500 g	0	0	8	6.1	13	3.1	0.023	NC		1.5	0.85–2.65	NC	
1500 g - 2499 g	5	19.2	16	12.1	91	21.6				0.59	0.36–0.95	1.46	0.62–3.45
>=2500 g	21	80.8	108	81.8	318	75.4				1.5		1.00	
Weight adequacy for GA													
Adequate for GA	19	76	110	83.3	312	75.4	0.0004	1.00		1.00		1.00	
Small for GA	5	20	15	11.4	97	23.4		0.85	0.33–2.23	0.51	0.31–0.85	1.7	0.71–4.03
Large for GA	1	4	7	5.3	5	1.2		2.90	0.46–18.31	2.24	1.34–3.71	0.85	0.13–5.56
5th minute Apgar													
<7	0	0	1	0.8	2	0.5	0.5603**			1.4	0.28–6.97		
>=7	25	100	131	99.2	418	99.5				1.00			
Neonatal disease													
Yes	8	30.8	27	21.3	122	29.4	0.0723	1.06	0.47–2.38	0.71	0.49–1.04	1.5	0.71–3.15
No	18	69.2	100	78.7	293	70.6		1.00		1.00		1.00	
Birth defect													
Yes	1	3.8	16	12.1	43	10.1	0.5135	0.37	0.05–2.67	1.16	0.74–1.82	0.33	0.05–2.30
No	25	96.2	116	87.9	382	89.9		1.00		1.00		1.00	
Anemia													
Yes	2	8.3	33	27.5	92	23.1	0.3183	0.32	0.08–1.33	1.2	0.85–1.69	0.28	0.07–1.14
No	22	91.7	87	72.5	307	76.9		1.00		1.00		1.00	
Thrombocytopenia													
Yes	3	12.0	6	5.0	13	3.3	0.0755	3.49	1.16–10.45	1.4	0.71–2.76	2.05	0.75–5.56
No	22	88.0	113	95.0	387	96.8		1.00		1.00		1.00	
Hepatic changes													
Yes	5	25.0	28	90.3	63	24.1	< 0.0001	1.04	0.39–2.77	20.62	6.43–66.07	0.18	0.08–0.42
No	15	75.0	3	9.7	198	75.9		1.00		1.00		1.00	
TOTAL	26		132		425								

*GA* gestacional age, *ATV* atazanavir, *NFV* nelfinavir, *LPV* lopinavir, *R* ritonavir

(#) Numbers are different due to the lack of information on some patients

(# #) Not calculated

*Chi-square, **Fisher Exact

Looking at the different protease inhibitors, the use of LPV increased the risk for low birth weight, specially between 1500 and 2499 g (*p* = 0.0230, RR 0.59 CI 0.36–0.95) and being small for the gestational age (SGA) (*p* = 0.0004, RR 0.51 CI 0.31–0.85) when compared to NFV use. NFV was associated with higher risk for liver function tests abnormalities (*p* < 0.0001, RR 20.62 CI 6.43–66.07) (Table 4).

**Table 4:** Comparative analysis of children exposed to tenofovir and zidovudine at CAISM/UNICAMP from 2000 to 2015

Variables	TDF		AZT		p*	RR	CI 95%
	*n*	%	*n*	%			
Gestational age (Capurro)					0.8962		
<37 weeks	8	21.1	153	22		0.95	0.44–2.03
>=37 weeks	30	78.9	544	78		1	
Birth weight					0.5221		
<2500 g	7	17.9	158	22.3		0.77	0.35–1.72
>=2500 g	32	82.1	550	77.7		1	
Weight adequacy for GA					0.3722	1	
Adequate for GA	28	73.7	563	80.3		1.29	0.60–2.76
Small for GA	8	21.1	123	17.5		2.48	0.64–9.59
Large for GA	2	5.3	15	2.1			
5th minute Apgar					1.000**		
<7	0	0	5	0.7			
>=7	38	100	701	99.3			
Neonatal disease					0.0056		
Yes	18	46.2	179	25.9		2.32	1.26–4.26
No	21	53.8	512	74.1		1	
Birth defect					0.0547**		
Yes	8	20	69	9.7		2.19	1.04–4.57
No	32	80	641	90.3		1	
Anemia					0.0030		
Yes	2	5.3	174	26.9		0.16	0.04–0.66
No	36	94.7	473	73.1		1	
Thrombocytopenia					0.6474**		
Yes	2	5.1	23	3.5		1.43	0.36–5.60
No	37	94.9	624	96.5		1	
Hepatic changes					0.0411		
Yes	19	51.4	101	34.2		1.86	1.02–3.41
No	18	48.6	194	65.8		1	
TOTAL	40		710				

*GA* gestational age, *TDF* tenofovir, *AZT* zidovudine

(#) The numbers are different due to lack of information about some patients

(##) Not calculated

*Chi-square, **Fisher Exact

Comparing TDF and AZT, the exposure to TDF was associated with higher occurrence of neonatal diseases (*p* = 0.0056, RR 2.32 CI 1.26–4.26) and increased liver function tests abnormalities (*p* = 0.0411, RR 1.86 CI 1.02–3.41), besides lower risk of anemia at birth (*p* = 0.0030, RR 0.16 CI 0.04–0.66) (Table 5).

**Table 5:** Multivariate analysis of risk factors associated with neonatal adverse effects

Risk Factors	Birth weight				Relative Risk	Logistic Regression	Gestational age at birth				Relative Risk	Logistic Regression	Anemia				Relative Risk	Logistic Regression	Hepatic alterations				Relative Risk	Logistic Regression
	<2500 g		>=2500 g				<37 weeks		>=37 weeks				Yes		No				Yes		No			
	*n*	%	*n*	%	RR CI 95%	RR CI 95%	*n*	%	*n*	%	RR CI 95%	RR CI 95%	*n*	%	n	%	RR CI 95%	RR CI 95%	*n*	%	*n*	%	RR CI 95%	RR CI 95%
Maternal age																								
<=28 yrs	84	47.2	332	54.1	1		102	54.0	312	52.2	1		104	56.2	270	50.2	1		63	49.2	104	46.2	1	
>28 yrs	94	52.8	282	45.9	1.24 (0.92–1.66)		87	46.0	286	47.8	0.95 (0.71–1.26)		81	43.8	268	49.8	0.84 (0.62–1.12)		65	50.8	121	53.8	0.93 (0.66–1.31)	
Hypertension																								
No	157	88.7	583	95.6	1		175	93.1	562	94.6	1		181	97.8	499	93.4	1		120	94.5	212	94.6	1	
Yes	20	11.3	27	4.4	2.01 (1.26–3.20)		13	6.9	32	5.4	1.22 (0.69–2.14)		4	2.2	35	6.6	0.39 (0.14–1.04)		7	5.5	12	5.4	1.02 (0.48–2.18)	
No information	1		4				1		4						4				1		1			
Diabetes during pregnancy																								
No	160	94.7	523	93.4	1		168	96.0	511	93.1	1		160	94.7	469	93.2	1		106	93.8	212	95.1	1	
Yes	9	5.3	37	6.6	0.84 (0.43–1.64)		7	4.0	38	6.9	0.63 (0.30–1.34)		9	5.3	34	6.8	0.82 (0.42–1.61)		7	6.2	11	4.9	1.17 (0.54–2.51)	
No information	9		54				14		49				16		35				15		2			
Coinfection during pregnancy																								
No	19	10.7	96	15.8	1		24	12.8	91	15.3	1		26	14.1	79	14.8	1		22	17.3	31	13.9	1	
Yes	158	89.3	513	84.2	1.42 (0.89–2.29)		163	87.2	503	84.7	1.17 (0.76–1.80)		159	85.9	454	85.2	1.05 (0.69–1.59)		105	82.7	192	86.1	0.86 (0.54–1.35)	
No information	1		5				2		4				0		5				1		2			
Smoking																								
No	88	77.2	362	87.7	1		104	85.2	341	85.3	1		115	90.6	288	83.2	1		70	83.3	61	69.3	1	
Yes	26	22.8	51	12.3	1.73 (1.12–2.67)		18	14.8	59	14.8	1 (0.57–1.65)		12	9.4	58	16.8	0.6 (0.33–1.09)		14	16.7	27	30.7	0.64 (0.36–1.13)	
No information	64		201				67		198				58		192				44		137			
Substance abuse																								
No	95	78.5	381	87.4	1		111	86.0	360	85.1	1		121	90.3	304	82.8	1		75	80.6	66	61.7	1	1
Yes	26	21.5	55	12.6	1.61 (1.04–2.48)		18	14.0	63	14.9	0.94 (0.57–1.55)		13	9.7	63	17.2	0.6 (0.34–1.07)		18	19.4	41	38.3	0.5 (0.34–0.96)	0.47 (0.27–0.83)
No information	57		178				60		175				51		171				35		118			
Alcoholism																								
No	98	92.5	388	94.9	1		113	95.8	368	93.9	1		123	96.9	312	92.9	1		79	95.2	69	81.2	1	
Yes	8	7.5	21	5.1	1.37 (0.67–2.81)		5	4.2	24	6.1	0.73 (0.30–1.80)		4	3.1	24	7.1	0.51 (0.19–1.37)		4	4.8	16	18.8	0.38 (0.14–1.02)	
No information	72		205				71		206				58		202				45		140			
First CD4 (cells/mm3)																								
<200	31	18.0	59	10.5	1		34	19.2	55	9.9	1		24	13.8	59	11.7	1		20	17.1	21	9.6	1	
>=200	141	82.0	505	89.5	0.64 (0.43–0.94)		143	80.8	499	90.1	0.58 (0.40–0.85)		150	86.2	444	88.3	0.87 (0.57–1.34)		97	82.9	198	90.4	0.67 (0.42–1.09)	
No information	6		50				12		44				11		35				11		6			
Peripartum CD4 (cells/mm3)																								
<200	30	17.5	47	8.36	1	1	32	18.2	44	8.0	1	1	21	12.1	50	9.9	1		18	15.5	18	8.2	1	
>=200	141	82.5	515	91.6	0.55 (0.37–0.82)	0.47 (0.29–0.77)	144	81.8	508	92.0	0.53 (0.36–0.77)	0.54 (0.37–0.79)	152	87.9	453	90.1	0.85 (0.54–1.34)		98	84.5	201	91.8	0.6 (0.40–1.08)	
No information	7		52				13		46				12		35				12		6			
Peripartum viral load																								
Undetectable (<50 copies/ml)	92	54.8	343	60.4	1		99	56.3	336	60.5	1		106	60.9	302	59.7	1		58	49.2	168	76.0	1	1
Detectable (>=50 copies/ml)	76	45.2	225	39.6	1.19 (0.88–1.62)		77	43.8	219	39.5	1.14 (0.85–1.54)		68	39.1	204	40.3	0.96 (0.71–1.31)		60	50.8	53	24.0	2.61 (1.44–2.97)	2.37 (1.52–3.68)
No information	10		46				13		43				11		32				10		4			
CDC classification																								
1	47	26.9	177	30.4	1		44	24.2	180	31.5	1		49	27.4	158	30.4	1		40	32.8	63	28.1	1	
2	84	48.0	304	52.1	1.03 (0.72–1.48)		87	47.8	298	52.2	1.15 (0.80–1.65)		91	50.8	266	51.3	1.08 (0.76–1.52)		54	44.3	114	50.9	0.83 (0.55–1.25)	
3	44	25.1	102	17.5	1.44 (0.95–2.17)		51	28.0	93	16.3	1.8 (1.20–2.70)		39	21.8	95	18.3	1.23 (0.81–1.87)		28	23.0	47	21.0	0.96 (0.59–1.56)	
No information	3		31				7		27				6		19				6		1			
Start of ART use																								
Before pregnancy	41	23.6	102	17.1	1		42	22.7	101	17.4	1		32	17.6	101	19.2	1		23	18.7	76	34.1	1	1
During pregnancy	133	76.4	495	82.9	0.74 (0.52–1.05)		143	77.3	480	82.6	0.78 (0.55–1.10)		150	82.4	424	80.8	1.09 (0.74–1.59)		100	81.3	147	65.9	1.74 (1.11–2.74)	1.5 (0.71–3.18)
No information	4		17				4		17				3		13				5		2			
Time of ART use during pregnancy																								
<=15 days	1	1.0	7	2.2	1		2	1.9	6	1.9	1		1	1.2	7	2.3	1		1	1.5	4	1.9	1	
>15 days	97	99.0	307	97.8	1.92 (0.27–13.77)		102	98.1	302	98.1	1.01 (0.25–4.09)		84	98.8	295	97.7	1.77 (0.25–12.73)		67	98.5	206	98.1	1.23 (0.17–8.84)	
No information	80		300				85		290				100		236				60		15			
NRTI																								
AZT	158	303.8	550	252.3	1		171	275.8	533	260.0	1		174	98.9	473	92.9	1	1	101	84.2	194	91.5	1	
TDF	7	13.5	32	14.7	0.8 (0.41–1.60)		6	9.7	32	15.6	0.65 (0.29–1.47)		2	1.1	36	7.1	0.2 (0.05–0.79)	0.21 (0.05–0.83)	19	15.8	18	8.5	1.5 (0.92–2.45)	
No information	13		32				12		33				9		29				8		13			
ART during pregnancy																								
NRTI+ NRTI + NVP	28	17.4	110	19.7	1		34	20.0	103	18.9	1		38	4.1	79	2.9	1		20	2.6	3	0.2	1	1
NRTI+ NRTI + NFV	24	14.9	108	19.4	0.9 (0.52–1.55)		28	16.5	102	18.8	0.87 (0.53–1.43)		33	3.6	87	3.2	0.85 (0.53–1.35)		28	3.6	3	0.2	1.04 (0.59–1.84)	0.95 (0.5–1.81)
NRTI+ NRTI + LPV/R	104	64.6	318	57.1	1.22 (0.80–1.84)		103	60.6	319	58.6	0.98 (0.67–1.45)		92	9.9	307	11.4	0.71 (0.49–1.04)		63	8.2	198	14.7	0.28 (0.17–0.46)	0.39 (0.19–0.79)
NRTI+ NRTI + ATV/R	5	3.1	21	3.8	0.95 (0.37–2.46)		5	2.9	20	3.7	0.81 (0.32–2.06)		2	0.2	22	0.8	0.26 (0.06–1.06)		5	0.7	15	1.1	0.29 (0.11–0.77)	0.48 (0.14–1.62)
No information	17		57				19		54				20		43				12		6			
Birht weight																								
>=2500 g													139	75.5	427	79.4	1		99	78.0	177	78.7	1	
<2500 g													45	24.5	111	20.6	1.18 (0.84–1.64)		28	22.0	48	21.3	1.03 (0.68–1.56)	
No information													1		0				1		0			
Gestational age at birth																								
>=37 weeks													126	68.5	428	80.0	1	1	104	81.9	171	76.3	1	
<37 weeks													58	31.5	107	20.0	1.55 (1.13–2.11)	1.44 (1.04–1.99)	23	18.1	53	23.7	0.8 (0.51–1.26)	
No information													1		3				1		1			
Mode of delivery																								
Vaginal	21	11.8	32	5.2	1.87 (1.18–2.94)	1.96 (1.09–3.52)	22	11.6	29	4.8	1.90 (1.22–2.97)	1.43 (0.85–2.39)	10	5.4	36	6.7	0.84 (0.45–1.59)		12	9.4	9	4	1.64 (0.90–2.96)	
Cesarean	157	88.2	582	94.8	1	1	167	88.4	569	95.2	1	1	175	94.6	502	93.3	1		116	90.6	216	96		
Preterm labor																								
No	134	75.7	541	88.7	1	1	123	65.4	551	92.8	1	1	151	81.6	466	87.3	1		115	90.6	187	83.5	1	
Yes	43	24.3	69	11.3	1.93 (1.37–2.73)	2.08 (1.34–3.24)	65	34.6	43	7.2	3.30 (2.44–4.45)	3.33 (2.43–4.54)	34	18.4	68	12.7	1.36 (0.94–1.98)		12	9.4	37	16.5	0.64 (0.36–1.17)	
No information	1		4				1		4				0		4				1		1			

*ART* antiretroviral therapy, *NRTI* nucleos(t)ide reverse transcriptase inhibitors, *AZT* zidovudine, *TDF* tenofovir, *NVP* nevirapine, *NFV* nelfinavir, *LPV* lopinavir, *ATV* atazanavir, *R* ritonavir, *CDC* Centers for Disease Control and Prevention

*Cox Logistic Regression

SGA children did not present higher occurrences of neonatal diseases (*p* = 0.1038), congenital malformation (*p* = 0.1902), anemia (*p* = 0.6578), liver function tests abnormalities (*p* = 0.9225) at birth or MTCT (*p* = 0.1759) when compared to children with adequate birth weight for gestational age (data not shown).

ART introduced prior to pregnancy was associated with higher preterm birth (*p* = 0.008) and with neonatal diseases (*p* < 0.0001). On the other hand, initiating ART during pregnancy was associated with higher instances of liver function tests abnormalities (*p* = 0.0024). There was no difference in the occurrence of congenital malformations regarding time of ART exposure during pregnancy (data not shown).

In multivariate analysis, there was no association between antiretroviral treatment regimen or time of ART exposure and preterm birth, low birth weight or birth defects. Compared to maternal AZT exposure, TDF use was less frequently associated with neonatal anemia (RR 0.21 CI 0.05–0.83). NVP use was associated with higher occurrence of neonatal liver function tests abnormalities (RR 0.39 CI 0.19–0.79), compared to maternal LPV/R exposure (Table 2).

## Discussion

This study analyzed 793 pregnancies of HIV infected women and 787 exposed newborns and showed high rates of neonatal adverse effects. Low birth weight occurred in 22.5%, preterm birth in 22%, SGA children in 18% and very low birth weight in 4% of all cases. Similar results were found in another Brazilian study with 74 children exposed to maternal ART between 2001 and 2012, where 34.8% were exposed since conception, with preterm birth rates of 17.5% and low birth weight of 20.2%, proportionally higher in women with AIDS [17].

In our cohort, preterm birth rates were much higher than another more recent multicentric study comprised of 20 reference hospitals in Brazil which showed a preterm birth rate of 12.3% for a general population of pregnant women, including women infected with HIV [18].

Contrary to our data, another study evaluating 214 HIV infected pregnant women in Rio de Janeiro between 2005 and 2006 showed low occurrence of preterm birth, low birth weight, malformations or obstetric complications [19].

Studies on preterm birth and low birth weight in children exposed to maternal HIV sometimes showed conflicting results, considering many factors (socioeconomic, maternal age, body mass index, ethnicity, smoking, substance abuse, multiple gestation, previous preterm birth, uterine infection and bacterial vaginosis) besides maternal diseases, genital and plasmatic viral load or ART exposure can increase the risk of preterm birth and low birth weight [20–22]. Most of the studies show high rates of preterm birth and low birth weight in children with maternal ART exposure, in line with our data.

In our study hypertension, smoking and substance abuse were associated with low birth weight while maternal AIDS were associated with preterm birth. On the other hand, in the multivariate analysis, only peripartum CD4 count lower than 200 cells/mm^3^ came up as a risk factor for preterm birth and low birth weight, and C-section as a risk factor for low birth weight but not for preterm birth. The association between severe immunosuppression and higher risk for preterm birth and low birth weight appears in most of the literature’s results [17].

C-section was associated with a higher occurrence of low birth weight since in situations of fetal grow restrictions there is a higher need for cesarean due to chronic fetal distress and low fetal reserves. The occurrence of C-sections was not associated with preterm birth since the institution’s protocol at that time included the recommendation for an elective C-section around 39 weeks of gestational age for patients infected with HIV to avoid iatrogenic prematurity. Nowadays, the institution guidelines recommend C-section only if the viral load is detectable after 35 weeks or for obstetrics’ reasons [23].

In our study, contrary to other published research, time of maternal ART exposure was not a risk factor for neonatal outcomes such as preterm birth and low birth weight. The increased risk for these two outcomes in children exposed to ART since conception was observed in a Latin America and Caribbean study of 1512 pregnant women and 1483 live births. It showed 19.8% preterm birth rates, 14.2% low birth weight, 12.6% SGA and 0.4% of neonatal deaths were associated with ART initiated prior to the pregnancy, smoking, low maternal body mass index, hospitalization and gestational hypertension [24]. A more recent cohort study from Tanzania which evaluated 3314 pregnant women infected with HIV also showed high rates of preterm birth (26%) associated with longer exposure to maternal drugs during pregnancy. The general rates for SGA was 21%, similarly to our study’s rate of 18%. Among the pregnant women which used PI regimens the preterm birth rate was 25% and the SGA was 13% [25] as in our study.

Data from a cohort of 9504 infected women in Botswana demonstrated that the HIV infection was associated with higher occurrence of stillbirth, preterm birth, SGA and perinatal death. Women in use of ART prior to pregnancy presented an even higher risk [26].

A study from Rio de Janeiro with 588 children exposed to maternal HIV between 1996 and 2010 showed an association between the use of ART in the first trimester and a higher occurrence of SGA, contrary to our findings [27].

Consistent with our data, a meta-analysis from 2007 showed no difference in instances of preterm birth among the various ART regimens. However, regimens with PI and ART use either before conception or early in pregnancy elevated the risk of preterm birth [28].

In our cohort, the different regimens of maternal ART evaluation demonstrated no association between AZT and TDF use with preterm birth or low birth weight. Nevertheless, a clinical trial (PROMISE trial) from six countries in sub-Saharan Africa and India showed an association between TDF exposure and higher rates of preterm birth and early neonatal death when compared to AZT exposure. Low birth weight and preterm birth were more frequent in the combined ART group compared to AZT monotherapy [29]. Data from the American Antiretroviral Pregnancy Registry from 1989 to 2013 also found an increased risk for low birth weight among patients utilizing AZT regimens [30].

In multivariate analysis of types of ART regimens, we found no association between a specific treatment regimen and higher occurrence of preterm birth and low birth weight, similarly to other studies [31]. We found no association of PI exposure and higher risk of low birth weight. It is currently considered that the maternal progesterone level reduction due to lower trophoblastic production in pregnant women using PI could be directly related to fetal weight and could explain preterm birth and low birth weight outcomes [32].

Various other studies also found no association between specific antiretroviral regimens and higher risk for low birth weight. This includes a study conducted from 2009 and 2013 in Zambia with 4474 women. They described low birth weight rates of 7%, less than in our study. They found no increased low birth weight in women using ART prior to or during pregnancy when compared to the ones that did not use it [33].

Our study showed no association between preterm birth and PI use despite the much higher preterm birth rates than those seen in the general population without HIV infection. This suggests that the infection itself can be a risk factor for preterm birth in these women. A study from Botswana compared the gestational outcomes in two different periods: 2009–2011 (when zidovudine monotherapy was recommended from the 28^th^ week with CD4 higher or equal to 350 cells/mm^3^ and combined ART for CD4 lower or equal to 350 cells/mm^3^) and 2013–2014 after the implementation of TDF/emtricitabine/EFV independently from CD4 count or the gestational week. Preterm birth rate was 22%, with no difference between the combined ART regimens, as in our results [34]. Another clinical trial compared LPV/R and EFV use in 356 infected pregnant women and found no difference in preterm birth rates between the groups [35].

The high complexity of maternal ART regimens was correlated with the increased risk of hematologic and hepatic adverse effects in most of the studies, including our cohort, which showed high rates of anemia and liver changes at birth. Of all the newborns, 25% presented with anemia and 3.6% with low platelet count. Most of the literature showed high rates of anemia in children exposed to antiretroviral maternal treatment. Consistent with our study, the one in Nigeria included 126 uninfected children exposed to maternal HIV and showed decreased in hemoglobin, hematocrits, leucocytes and neutrophils in children exposed to both, maternal HIV and antiretroviral drugs [36]. Although many studies evaluated occurrence of anemia comparing monotherapy with AZT and combined ART, in our study the small number of cases using this regimen (only 23 cases with monotherapy) prevented the comparison. In our multivariate analysis, anemia at birth was associated with exposure of regimens containing AZT (comparing with TDF) and to preterm birth.

Another study analyzing adverse hematologic effects in 221 uninfected children exposed to maternal ART showed 54% of anemia and 40% neutropenia. ART with NNRTI or PI presented as an independent risk factor for anemia and neutropenia during the first three months of life in comparison to monotherapy or double therapy. No significant low platelet count was found but 60% of the children presented with thrombocytosis [37]. Likewise, no significant evidence of low platelets was found in our study.

Like our results, a recent European cohort study on children exposed to maternal ART in their first year of life compared the two periods of exposure (2000–2001 and 2007–2013). It demonstrated lower occurrence of anemia and neutropenia in the second period, with higher frequency of adverse effects when using regimens containing AZT [38].

There are some studies that also showed adverse effects associated with neonatal prophylaxis. An American study followed 147 uninfected children from 1997 to 2009 compared neonatal prophylaxis with monotherapy with AZT and triple therapy (the majority with AZT/3TC/NVP). The adverse effects with combined 3-drugs therapy and monotherapy with AZT were respectively: neutropenia 55% and 39%; anemia 50% and 39%; low platelet count 0 and 3%; elevation in AST 3% and 3%; elevation in ALT 0 and 1%; hyperbilirubinemia 19% and 42%. Anemia occurred more frequently in children who received prophylaxis with combined ART than in the ones who received AZT monotherapy. Despite the adverse effects being typical of AZT toxicity, the combination of AZT/3TC/NVP increased the frequency of severe anemia [39]. Brazilian guidelines do not recommend the combined regimen of three drugs for neonatal prophylaxis. In our cohort, every child received neonatal AZT. Because in our study we had only 24 children using the association between NVP and AZT in the first week of life as neonatal prophylaxis in women with detectable viral load at delivery, this comparison was not performed.

Hepatic changes at birth was a frequent adverse effect in our cohort (36%) associated mostly with NVP exposure. Consistent with our results, one recent American study showed that children exposed to lopinavir/ritonavir presented lower incidence of hematological and hepatic changes [40]. However, in other studies no association was found between hepatic change and specific drug regimens, including a Brazilian study [41]. Studies from Kenya and Spain showed no difference between exposure to NVP and NFV for hepatic toxicity [42, 43].

One limitation in our study was lack of follow-up of children preventing the MTCT diagnosis confirmation. Despite the many efforts to contact the families through phone, children were not brought to the clinic for re-testing. Testing children for HIV viral load at birth to detect intrauterine infection is not recommended in the national guidelines although it could have contributed to diagnosing MTCT. Another limitation was not systematically collecting serum lactate levels in the newborns to evaluate mitochondrial toxicity although, clinically, no signs of this event was observed. Our study used LPV/R more frequently than the others ART regimens making it more difficult to compare their effects. However, the goal of our study was to describe each antiretroviral adverse effect in this population to define the best regimen to reduce MTCT and adverse effects.

## Conclusions

In general, we observed that adverse effects in children exposed to HIV maternal antiretroviral treatment were too frequent but not severe and MTCT rates were low independently from the antiretroviral regimen, reinforcing the importance of adequate maternal treatment and total viral load control. The huge benefit of preventing HIV MTCT will always take precedent over low severity of adverse effects such as observed in our study.

Changes in maternal therapy recommended by Brazilian guidelines, using a new class of drugs - integrase inhibitors - as first line regimens for adults and pregnant women, will demand future evaluation on its benefits in preventing HIV MTCT as well as maternal and neonatal adverse effects.

## Abbreviations

3TC: Lamivudine
AIDS: Acquired immunodeficiency syndrome
ALCPH: Alkaline phosphatase
ALT: Alanine aminotransferase
ART: Antiretroviral therapy
AST: Aspartate aminotransferase
ATV: Atazanavir
AZT: Zidovudine
CAISM/UNICAMP: Women’s Hospital at University of Campinas School of Medical Sciences
CI: Confidence interval
DRV: Darunavir
EFV: Efavirenz
GGT: Gamma-Glutamyl Transferase
HIV: Human immunodeficiency virus
LPV: Lopinavir
MTCT: Mother-to-child transmission
NFV: Nelfinavir
NNRTI: Non-nucleoside reverse transcriptase inhibitor
NRTI: Nucleoside reverse transcriptase inhibitors
NVP: Nevirapine
PI: Protease inhibitor
R: Ritonavir
RAL: Raltegravir
RR: Relative risk
SGA: Small for gestational age
TDF: Tenofovir
WHO: World Health Organization
